# Multi-omics in Allergic Rhinitis: Mechanism Dissection and Precision Medicine

**DOI:** 10.1007/s12016-025-09028-3

**Published:** 2025-02-18

**Authors:** Yan Hao, Yujuan Yang, Hongfei Zhao, Ying Chen, Ting Zuo, Yu Zhang, Hang Yu, Limei Cui, Xicheng Song

**Affiliations:** 1 Shandong University of Traditional Chinese Medicine, Jinan, 250000 Shandong China; 2 Qingdao Medical College, Qingdao University, Qingdao, 266000 Shandong China; 3 Department of Otolaryngology Head and Neck Surgery, Yantai Yuhuangding Hospital, Qingdao University, Yantai, 264000 Shandong China; 4 Shandong Provincial Clinical Research Center for Otorhinolaryngologic Diseases, Yantai, 264000 Shandong China; 5 Yantai Key Laboratory of Otorhinolaryngologic Diseases, Yantai, 264000 Shandong China; 6 The 2Nd Medical College of Binzhou Medical University, Yantai, 264000 Shandong China

**Keywords:** Allergic rhinitis, Genomics, Proteomics, Precision medicine

## Abstract

Allergic rhinitis (AR) is a common chronic inflammatory airway disease caused by inhaled allergens, and its prevalence has increased in recent decades. AR not only causes nasal leakage, itchy nose, nasal congestion, sneezing, and allergic conjunctivitis but also induces asthma, as well as sleep disorders, anxiety, depression, memory loss, and other phenomena that seriously affect the patient’s ability to study and work, lower their quality of life, and burden society. The current methods used to diagnose and treat AR are still far from ideal. Multi-omics technology can be used to comprehensively and systematically analyze the differentially expressed DNA, RNA, proteins, and metabolites and their biological functions in patients with AR. These capabilities allow for an in-depth understanding of the intrinsic pathogenic mechanism of AR, the ability to explore key cells and molecules that drive its progression, and to design personalized treatment for AR. This article summarizes the progress made in studying AR by use of genomics, epigenomics, transcriptomics, proteomics, metabolomics, and microbiomics in order to illustrate the important role of multi-omics technologies in facilitating the precise diagnosis and treatment of AR.

## Introduction

Allergic rhinitis (AR) is a chronic inflammatory condition that affects the nasal mucosa and is caused by immunoglobulins (IgE). At the beginning of the twentieth century, AR was considered a rare disease of the wealthy; however, by the latter part of the twentieth century, the prevalence of AR began to increase exponentially and is now one of the most prevalent chronic diseases [1]. A global study of > 1 million adolescents showed an AR prevalence as high as 14.6% [2], and almost half of the patients with AR displayed symptoms before they were 6 years old. Typical symptoms include nasal leakage, itchy nose, nasal congestion, sneezing, and allergic conjunctivitis, which can last for several years [3]. There is an obvious epidemiologic link between AR and asthma, and asthma is likely to be triggered if AR is left untreated [4]. In addition, approximately 66% of AR patients have experienced a sleep disorder [5], which may cause brain-related symptoms such as memory loss, anxiety, despair, and diminished sense of smell, all of which negatively impact a person’s quality of life and place a significant burden on the patient’s family and society [6].

**Fig. 1:**
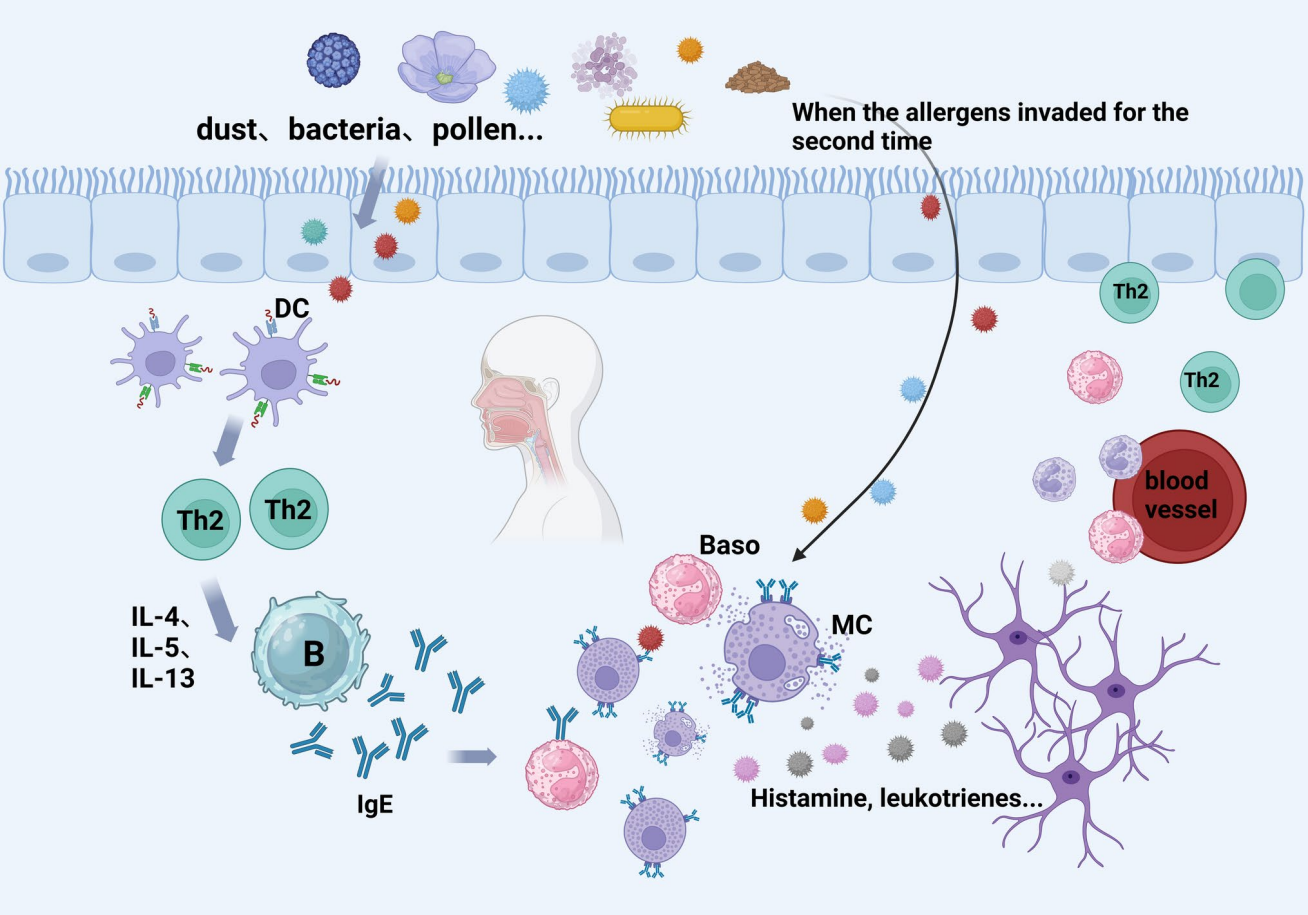
Pathogenesis of allergic rhinitis. The allergic reaction process. Allergens such as dust, bacteria, and pollen enter the body. Dendritic cells (DC) recognize them and activate Th2 cells. Th2 cells produce cytokines that stimulate B cells to make IgE antibodies. When allergens re-enter, they bind to IgE on mast cells and basophils, causing those cells to release histamine and leukotrienes, leading to an allergic reaction

Healthy epithelial cells in the airways protect the host from environmental hazards, but when compromised, those cells may activate immune-inflammatory responses against exogenous allergens, environmental microorganisms, and pollutants, triggering disorders such as AR [7]. When an allergen enters the body for the first time, antigen-presenting cells transmit it to Th cells, which become activated and secrete cytokines such as IL-4 and IL-13. Those cytokines act on sIgG⁺B cells to reconstitute and produce sIgE at the mucosal level. SIgE binds to the Fc receptor on the surface of mucosal mast cells and basophils and completes sensitization to the allergen [8]. Upon re-exposure to the same allergen, allergens that have cross-linked with cell surface-bound IgE attach to neighboring IgE molecules, triggering the degranulation of mast cells and basophils. Additionally, a rapid release of histamine and other inflammatory mediators occurs, which leads to smooth muscle contraction, vasodilation, and recruitment of inflammatory cells. These events produce symptoms such as sneezing, nasal congestion, and runny nose [9]. Localized sIgE in the mucous membranes plays a key role in the local allergic inflammatory response, and a variety of cytokines and immune cells interact with each other to influence IgE synthesis and allergic processes (Fig. 1).

Currently, AR is treated with pharmacological therapy as well as immunotherapy. Pharmacologic treatments mainly include antihistamines and intranasal corticosteroid nasal sprays; however, those drugs only provide a temporary relief of symptoms [10–13]. Immunotherapy mainly includes the use of specific biologic agents. Specific immunotherapy, which involves injecting or giving small doses of a particular allergen to a patient at regular intervals to gradually build up tolerance to that allergen, can be given in the form of subcutaneous injections or sublingual drops and has been demonstrated to offer long-term improvement of AR symptoms and reduce a patient’s dependence on medications [14]. Anti-IgE antibodies (e.g., omalizumab) can be used in patients with severe AR [15]. Currently, omalizumab is the only globally approved therapeutic anti-IgE biologic agent and is approved for use in Japan for severe AR that has failed to respond to pharmacologic and non-pharmacologic interventions. By comparing a treatment group using omalizumab with a placebo group, it was found that omalizumab significantly reduced a patient’s itchy, sneezy, and runny nose, which are typical AR symptoms that occur during the Japanese willow pollen season. Several randomized controlled trials have shown that treatment with omalizumab significantly reduces a patient’s rhinitis symptom score, alleviates symptoms, and reduces the use of emergency medications [16].

The accurate diagnosis of AR presents many dilemmas. In terms of assessing the clinical relevance of IgE sensitization, both serum-specific IgE tests and skin prick tests have shortcomings, as a positive test does not necessarily identify the causative allergen, and IgE levels do not simply correspond to disease severity, which can be complicated by sensitization to multiple allergens. It is even more difficult to identify localized AR in non-atopic patients. Serum sIgE is often negative, and there is a lack of specific diagnostic markers. Localized inflammation in the nasal cavity is very similar to that which occurs in non-AR, which is difficult to differentiate from non-AR in terms of both symptoms and pathological changes. Furthermore, it is difficult to distinguish AR by using conventional diagnostic techniques, which can easily lead to its misdiagnosis or omission of diagnosis. The complexity and diversity of AR etiology make it difficult to accurately recognize and avoid allergens and make treatment difficult [17]. Additionally, the large individualization of patients, the poor response of some patients to conventional treatments, and the lack of specific markers and effective targets greatly limit the early diagnosis of AR and the ability to develop a personalized treatment.

Data from transcriptomics, proteomics, metabolomics, genomics, and epigenomics are all integrated in multi-omics research to form a complete biological information flow from genes to metabolites. This information flow starts with the coding of genes and then progresses to the regulation of gene expression, execution of protein function, and finally to the impact of metabolic reactions, in a hierarchical manner. Multi-omics can analyze the complexity of organisms in an all-encompassing manner, reveal the intrinsic mechanisms of organisms in both healthy and diseased states, identify new biomarkers and potential therapeutic targets, and provide more precise and comprehensive diagnostic and therapeutic strategies for AR. This article outlines the progress made in using genomics, epigenomics, transcriptomics, proteomics, and metabolomics for studying AR. It also summarizes, integrates, and analyzes data obtained from previous studies to demonstrate the significant contribution that multi-omics technology makes to advancing the accurate diagnosis and treatment of AR and provides a fresh approach to the accurate diagnosis and care of individuals suffering from this condition.

## Advances in the Use of Multi-omics in AR

### Genomics

Genomics provides a comprehensive blueprint of all genetic information for a species, including the identification of genes, genome structure and organization, genetic variation, and genome evolution. It is well known that changes in DNA sequence can significantly increase the risk for developing a wide range of rare and common diseases, and genetic testing technologies are increasingly being incorporated into medical practice [18, 19]. Since 2005, initial cataloging of common DNA single nucleotide polymorphisms has led to the beginning of commercial microarray design [20]. Researchers have attempted to link single nucleotide polymorphisms to specific diseases by performing genome-wide association studies. The emergence of high-throughput sequencing in the late 1920s, which is capable of simultaneously sequencing millions of DNA molecules, has allowed scientists to perform both whole-exome sequencing and whole-genome sequencing, which has dramatically increased sequencing efficiency [21]. Because DNA sequences in individual non-cancer somatic cells are relatively stable, genome-wide variations among individual sequences can be quantified by extracting high-quality DNA from a single sample of peripheral blood or saliva collected at any time during an individual’s lifetime [22]. Due to the cost-effectiveness of genomics research, the stability of the technology, the simplicity of collecting samples, and the generalizability and stability of DNA sequences in human cells over a lifetime, genomics has become more commonly used, with sample sizes far exceeding those of other omics approaches.

#### Research on Systemic Samples

Genomic studies have found a genetic association regarding AR pathogenesis. According to estimates, AR has a high hereditary component, with a heritability index > 0.65 [23]. Several reports have linked genetic loci and candidate genes to AR [24–26]. Evidence for the heritability of AR was provided by a twin study of twin families using data obtained from the Dutch Twin Registry, which was established by the Department of Biological Psychology at the Free University of Amsterdam. AR was found to occur in approximately 45 to 60% of those identical twins at the same time, while in fraternal twins, the probability declined to 25% [27]. In order to clarify the genetic structure and pathogenesis of AR, researchers conducted a global association study in a cohort of individuals with European ancestry and analyzed 59,762 patients with AR and 152,358 control subjects. The study identified 41 loci associated with the risk for AR, including 20 newly discovered sites. Next, the loci were verified during a replication step that involved 60,720 patients and 618,527 controls [28]. For example, gene *IL7R* on chromosome 5p13.2, gene *SH2B3* on chromosome 12q24.12, and chromosome 19q13.11 were found to be possibly associated with AR [29–31]. AR also shares a partially common genetic origin with asthma and atopic dermatitis [32]. Various causative genes of AR (e.g., *SDAD1*, *CXCL10*, and *CXCL9*) have been found to be related to an array of immune-related disorders [33]. Those genes affect common genetic risk variants for atopic diseases, which implies that they can display typical pathogenic traits and be used for creating novel treatments [34]. Those investigations also demonstrated the close association between AR and autoimmune and allergy disorders and provided clues for understanding the pathophysiology and genetic makeup of AR, as well as a foundation for its accurate diagnosis and treatment (Table 1).

**Table 1:** Synopsis of the genetic loci linked to allergic rhinitis

Chromosome	Gene	Possible allergic mechanism
4q27	*IL2*	Immune regulatory effects [25]
16p13.13	*CLEC16A*	Highly expressed in lung T and B cells [35]
5p13.2	*CAPSL*;* IL7R*	B and T cell receptors undergo V(D)J recombination. The amount of IL-7R on the cell surface varies among T cell subtypes [28]
12q24.31	*CDK2AP1*;* C12orf65*	Involved in hematopoiesis [28]. Involved downstream of T cell receptor activation [28]
6q15	*BACH2*;* GJA10*	Antigen-induced generation of memory T cells and memory B cells [28]
11q23.3	*CXCR5*;* DDX6*	B cells express chemokine receptors, which play a role in B cell migration in the lymph nodes and spleen. *CXCR5* is expressed on a subset of follicular T cells [28]
15q15.1	*ITPKA*;* RTF1*	*TYRO3* stimulates *SOCS1* and suppresses TLR-mediated immunological signaling. T cell receptor activation is followed by involvement of leukocyte tyrosine kinase [28]
4q24	*MANBA*;* NFKB1*	*NFKB1* mediates signals from TLRs and cytokines and activates inflammatory pathways [36]
7p15.1	*JAZF1*;* TAX1BP1*	Transcriptional repressor in Th2 diabetes, multiple sclerosis, and endometrial stromal tumors [28]
12q24.12	*ATXN2*;* SH2B3*	Downstream of the activation of T cell receptors and involved in hematopoiesis [28]
9q34.2	*ABO*; *OBP2B*	Allele variants of *ABO* determine blood group type [28]
3p21.2	*VPRBP*	Participates in V(D)J recombination and T cell proliferation during B cell development [28]
2p23.2	*FOSL2*; *RBKS*	Cell cycle and proliferation [28]
10q24.32	*ACTR1A*;* TMEM180*	Subunit of the NFKB complex and involved in regulating TLR-4 and cytokines [28]
1p31.1	*LRRIQ3*; *NEGR1*	Cell adhesion [28]
19q13.11	*CEBPA*; *SLC7A10*	*CEBPG* interacts with immunoglobulin heavy chain transcriptional enhancers. Lung development and inflammatory bowel illness are also affected by *CEBPA* [37]
12q24.31	*SPPL3*; *ACADS*	*OASL* is involved in IFN-γ signaling. Regulates the number of NK cells [28]
2q36.3	*SPHKAP*; *DAW1*	Dynein assembly factor [28]
15q22.2	*RORA*	Involved in the development of natural helper cells [38]
1p36.23	*RERE*; *SLC45A1*	Apoptosis-associated transcription factor [39]
13q14.11	*TNFSF11*;* AKAP11*	Assists dendritic cells to enhance T cell activation [28]

Numerous studies have confirmed that rural farm environments, transportation pollution, tobacco smoke, climate change, pet ownership, and occupational factors are associated with IgE levels and allergic diseases [40–43]. The rural farm environment is negatively associated with IgE levels and allergic diseases, while traffic pollution and tobacco smoke are positively associated with IgE levels and allergic diseases. Other factors depend on specific circumstances. In a nested case-control study of 400 Swedish children, researchers found that lower floors of a building were more susceptible to moisture and mold when compared to higher floors, which may increase a person’s susceptibility to AR [44]. In children with AR, Chen et al. [45] reported a gene-environment association between moldy scents and the polymorphism rs769214. The relationship between pet ownership and AR is not static and can be affected by multiple factors, such as the type of pet, the environment in which the pet is kept, and the pet owner’s allergy [46]. Research has demonstrated that children and adolescents who are regularly exposed to dogs have higher blood levels of both specific and total IgE due to the CD14rs2569190 polymorphism [47]. In a case study of 237 AR patients, a logistic regression analysis showed that gene-environment interactions may have an impact on when AR first occurs [26]. In summary, a good living environment is essential for AR prevention.

Pharmacogenomics is another important area of genomic research in AR. It involves the study of causal alterations associated with a patient’s response to AR medications and/or susceptibility to adverse effects. To better understand the mechanisms and key genes involved in allergen immunotherapy (AIT) for AR, Fan et al. [48] used gene expression datasets (GSE37157 and GSE29521) to analyze differentially expressed samples obtained from allergic patients before AIT and samples from allergic patients undergoing AIT. The analysis identified 119 co-upregulated differentially expressed genes and 33 co-downregulated differentially expressed genes. A total of 20 pivotal genes were obtained from the PPI network, and those genes represented possible new targets for AIT in AR. The effects of AIT in AR were found to be reliably predicted by *CASP3*, *FOXO3*, *PIK3R1*, *PIK3R3*, and *ATF4.* Pharmacogenomic research can assist in identifying novel targets and treatment approaches for AR immunotherapy.

Genomics plays a significant role in studying AR. Genetically, AR is highly heritable, and genome-wide association studies have identified numerous risk loci and common genetic origins with other immune-related diseases, thereby providing a basis for diagnosis and treatment. Environmental factors, such as rural farm environments, are associated with AR pathogenesis, and gene-environment interactions are known to exist. Pharmacogenomic studies have identified potential targets for AR immunotherapy, which could help to develop new treatment strategies. However, there are limitations to using genomics to study AR. For example, there is great uncertainty about the relationship between pet ownership and AR. Furthermore, the potential targets identified by pharmacogenomics are not ready for clinical application, and in-depth validation studies are needed to realize the application of precision medicine in treatment of AR.

### Epigenomics

Epigenomics refers to the study of epigenetic alterations at the genomic level based on changes in gene expression resulting from non-genetic sequence alterations. This area of research focuses on mechanisms that regulate gene expression, and the interactions of environmental genetic factors, such as DNA methylation and histone modification. Similar in concept to genome-wide association studies, epigenomic association studies quantify the relationships between different illnesses or phenotypes and epigenetic changes throughout the genome [49]. Epigenetic studies of AR have focused on several core aspects covering the regulation of gene expression, DNA methylation, histone modifications, and the role of non-coding RNAs.

#### Research on Systemic Samples

Research has demonstrated a correlation between the quantity and pattern of CD4 + T cells in AR and the DNA methylation pattern, which can be used to differentiate allergic patients from healthy individuals. Researchers measured the expression levels of IL-10, TGF-β, and IFN-γ in the serum of patients with AR by ELISA and eventually found that hypermethylation of DNA led to a decrease in IFN-γ expression [50]. A total of 60 AR patients and 65 control subjects were enrolled by Li et al. The status of DNA methylation in peripheral blood leukocytes was screened using the MassARRAY EpiTYPER and pyrophosphate sequencing systems, and DNA hypomethylation was found to increase IL-33 and IgE mRNA expression [51]. Both the expression level of *SLFN12* and its DNA methylation status are important biomarkers for predicting the severity of allergic reactions. A low level of *SLFN12* expression might be attributed to a large amount of DNA methylation, and such changes in methylation patterns may be one of the mechanisms leading to an exacerbation of allergic symptoms [52]. During dual sublingual immunotherapy (SLIT) in patients with respiratory allergies, the therapeutic effect of treatment was found to be correlated with the degree of DNA methylation at the CpG site within the *Foxp3* gene locus, which is a specific genetic marker in memory-regulatory T cells. Compared with control subjects treated with placebo, the subjects who received dual SLIT had lower rhinoconjunctivitis scores and medication use scores, reduced responses to allergens, and decreased DNA methylation levels at CpG sites within the *Foxp3* locus. Epigenetic modification of *Foxp3* in memory-regulatory T cells may induce long-term tolerance of an organism to allergens, thereby enhancing a drug’s therapeutic efficacy [53]. DNA methylation is involved in the progression and therapeutic efficacy of AR in multiple ways.

#### Research on Local Samples

MicroRNAs are non-coding RNAs that are crucial for controlling inflammatory processes and considered to be promising biomarkers. Suojalehto et al. [54] analyzed inflammatory cells, cytokines, and miRNAs in nasal biopsies from 117 AR patients and found that miR-498, miR-187, miR-874, miR-143, and miR-886-3p levels were upregulated in AR patients, while miR-18a, miR-126, let-7e, miR-155, and miR-224 levels were downregulated. In addition to being a component of epigenetic machinery, miRNAs also cause DNA methylation and histone modifications, which are epigenetic modifications similar to those found in protein-coding genes [55]. By controlling other epigenetic regulators and altering the expression of genes that code for proteins by attracting particular protein complexes to particular promoters, miRNAs have an impact on the genome at different levels. Fabbri et al. [56] observed for the first time that enforced expression of miR-29s in lung cancer cell lines restored the normal pattern of DNA methylation by causing methylation-induced silencing of tumor re-expression of tumor suppressor genes (e.g., *FHIT* and *WWOX*) and inhibited tumorigenicity both in vitro and in vivo. These findings support the role of miR-29s in the epigenetic normalization of NSCLC and provide a theoretical basis for studying the roles played by miRNAs in epigenetics.

In addition, histone acetylation also affects the development of AR; histone deacetylase (HDAC) in immune cells is upregulated in nasal epithelial cells, and when HDAC is inhibited, the symptoms of AR improve [57]. HDAC1 inhibitors such as troglodyte inhibitor A and sodium butyrate were demonstrated to alleviate nasal epithelial dysfunction in mice [58]. Numerous other studies have proven a significant decrease in TWIK-associated potassium channel-1 (TREK-1) expression in AR, and that TREK-1 expression in nasal mucosa can be increased and HDAC1 levels decreased by antigen-specific immunotherapy, suggesting that an increase in HDAC1 may inhibit TREK-1 expression and thereby exacerbate AR symptoms [59–61]. Further studies found that inhibition of HDAC1 not only elevated the expression of anti-inflammatory cytokines such as IL-10 and Foxp3, and thereby prevented the over-activation of immune cells, but also reduced the production of the inflammatory cytokine, TNF-α [62]. Those results suggest that increased HDAC activity may contribute to AR development by altering the balance between pro- and anti-inflammatory cytokines.

Epigenetic factors offer several potential treatment methods for AR. In terms of DNA methylation, it is possible to predict the efficacy of immunotherapy by detecting the methylation of specific genes and adjusting the treatment regimen accordingly. Methyltransferase inhibitors or activators can also be used to regulate the methylation level of genes. For microRNA-related strategies, miRNA mimics or antagonists can be employed to adjust their levels, and drugs targeting the related pathways can be developed. Regarding histone acetylation, HDAC inhibitors can improve AR symptoms and regulate the cytokine balance. While further optimization and application studies are needed, the use of HDAC inhibitors in combination with existing AR treatment drugs can also be considered. Regulation of the epigenetic state via multiple approaches is expected to provide new directions and enhance the efficacy of AR treatment.

### Transcriptomics

Transcriptomics is the study of all types of RNA molecules, including coding RNAs (mRNAs), as well as non-coding RNAs such as miRNAs and lncRNAs. It allows for comparisons of cells or tissues in a disease state or under controlled conditions to identify significant changes in the expression of genes or transcripts. Identifying such changes can lead to the discovery of new biomarkers or the development of a specific hypothesis regarding cellular function. Transcriptomics involves quantitative and functional studies of RNA molecules and also investigations of how they are transcribed, processed, edited, and degraded [63]. Transcriptomics creates an inevitable link between genomic genetic information and biological functions. Compared to whole-genome sequencing of eukaryotes, the sequences obtained by transcriptome sequencing do not contain introns and other non-coding sequences; therefore, transcriptome sequencing has an incomparable cost-effective advantage. The study of transcriptomics has helped scientists to understand how disease-causing genes in AR are regulated in different environments, at different developmental stages, and in disease states.

#### Research on Systemic Samples

To identify the phenotypes and underlying mechanisms of childhood AR, Youn et al. [64] analyzed and clustered 1050 samples of blood obtained from children and used the samples for transcriptomic studies. The results revealed five clinical phenotypes of AR: early-onset (*n* = 88, 8.4%), moderately transient (*n* = 110, 10.5%), late-onset (*n* = 209, 19.9%), very late-onset (*n* = 187, 17.8%), and never/infrequent type (*n* = 456, 43.4%). The very late and late-onset AR phenotypes were positively associated with inhaled allergens and bronchial hyper-responsiveness in asthma, whereas the early-onset phenotypes were not associated with bronchial hyper-responsiveness. Airway hyper-responsiveness is a determinant of the early asthmatic response to an inhaled allergen [65]. By using microarray datasets for asthma and AR obtained from the Gene Expression Omnibus (GEO) database established by the National Center for Biotechnology Information (https://www.ncbi.nlm.nih.gov/geo/), Wang et al. [66] performed transcriptomic analyses which showed that CST1 was the only upregulated factor in patients with AR and asthma. Upregulated genes in the upper and lower airways may be indicators of airway allergy disorders and are important in the development of co-morbid asthma in AR. Additional research should be conducted on targeted therapy for CST1, as it may help AR patients avoid developing asthma. Peng et al. [67] used circRNA microarrays to screen peripheral blood samples from AR patients and healthy controls for circRNAs that were differently expressed. Those results showed that circRNA_404013 expression was significantly upregulated in AR. CircRNA_404013 may play a role in AR by regulating the level of brain-derived neurotrophic factor expression by affecting miR-182-5p. A study by Zhou et al. [68] focused on the lncRNA and mRNA expression profiles of monocyte-derived dendritic cells, pointing out that the study of lncRNAs should not be confined to T cells, and emphasizing the importance of co-expression of multiple cell types and multiple transcriptional loci in the pathogenesis of AR. Yin et al. [69] employed single-cell RNA sequencing to comprehensively investigate the transcriptional changes in B cells among peripheral blood mononuclear cells from patients with AR and identified factors that influence the differentiation of B cell immunoglobulins (mainly, IGHE, IGHGs, IGHA, IGHD, and IGHM). The results suggested ITGB1 as a potential predictive marker for AR patients who have received endocervical lymphoid immunotherapy. Sviatlana et al. [70] explored the immunomodulatory mechanisms of Pollinex Quattro Grass AIT in AR based on transcriptomic profiling of peripheral blood mononuclear cells. Transcriptomic profiles of peripheral blood mononuclear cells revealed the suppression of Th2 and Th17 pro-inflammatory allergic responses by Pollinex Quattro Grass AIT, as well as immune deflection towards Th1 responses.

#### Research on Local Samples

Suojalekto et al. [71] analyzed the transcriptomics of nasal mucosa from patients with AR, AR plus asthma, non-AR, and healthy controls. The data showed that miR-498, miR-155, and miR-205 were upregulated in the AR group, whereas the levels of let-7e miRNA were downregulated in AR, and those changes were associated with elevated levels of Th2 cytokines. When members of the let-7 family are elevated, they activate the JAK1/STAT3 signaling pathway, inhibit IL-13 production, and repress *SOCS4* gene transcription, which in turn suppresses the expression of various inflammatory factors in AR [72]. Wu et al. [73] isolated cells from the nasal mucus from healthy control subjects (*n* = 10) and patients with severe AR (*n* = 10). The extracellular vesicles were analyzed for vesicular RNA using miRNA probes, and selected results were validated by quantitative RT-PCR. A total of 21 miRNAs were found to be upregulated, and 14 miRNAs were found to be downregulated in the AR vesicles as compared with the vesicles from healthy control subjects, with one of the most significantly upregulated molecules being miR-223. When considering that miR-223 plays a role in eosinophilic inflammation and is associated with lower numbers of regulatory T cells, mirR-223 could be utilized as an adjuvant treatment during immunotherapy [74]. Researchers have conducted many studies on various miRNAs in AR patients, and we have summarized the functions and targets of AR-specific miRNAs in recent years (Table 2) [75]. Through further research and application of these targets, the role of miRNAs in AR will be more comprehensively understood and translated into practical diagnostic and therapeutic tools that can be used in precision medicine for AR. Wei et al. [76] investigated circRNA_404013 expression in the nasal mucosal tissues of patients with AR and healthy control subjects by performing a genomic analysis of lncRNAs. The results identified 57 differentially expressed lncRNAs; among which, 22 were upregulated and 35 were downregulated. GO and KEGG enrichment analyses revealed pathways such as endoplasmic reticulum, protein export, MAPK signaling pathway, and protein processing in leukocyte transformation and migration, suggesting that lncRNAs may play a key role in the pathophysiological process of AR. In particular, the researchers pointed out that leukocyte chemotaxis may be a potential target of lncRNA regulation in AR. Yang et al. [77] conducted a genome-wide analysis of lncRNA expression in nasal mucosal tissues by microarray detection and confirmed the co-expression relationship between lncRNAs and mRNAs. Those findings suggest that the pathogenesis of AR involves the co-regulation of lncRNAs and mRNAs and emphasize the roles of transcription factors Oct-1, AP-1, NF-κB, and c-Rel in the lncRNA-mediated AR mechanism.

**Table 2:** The functions and targets of AR-specific miRNAs based on existing research

miRNA	*Target*	Function
let-7	*SOCS4*, *JAK1/STAT3*,* IL-13*	Regulating the release of IL-13 and managing Th2 inflammation [72, 78]
miR-206	*S100A7A*, *VEGF*	Involved in the *VEGF* pathway [79]
miR-338-3p	*WNT/β-Catenin*	Inhibition of the *Wnt/β-catenin* pathway to induce the epithelial-mesenchymal transition [79]
miR-16	*IκB/NF-κB*	Inhibits the release of inflammatory cytokines induced by IL-13 [80]
miR-498	*STAT3*	Suppresses Th17 cell differentiation [54, 78]
miR-187	*CD276*	Regulation of the T cell response [54, 81]
miR-143	*TGF-β1*	Modulates memory T cell differentiation [54, 81]
miR-886-3p	*SMAD3*,* FoxO1*	Regulates the TGF pathway via *SMAD3* [54, 81]
miR-224	*SMAD4*	Regulates the TGF pathway via *SMAD4* [54, 81]
miR-155	*IL-13*	Controls the *IL-13* pathway in macrophages and the growth and differentiation of Treg cells [71, 82]
miR-126	*VEGF*, *IRS1*	Decreased expression in mononuclear leukocytes, and helps to regulate IL-4 [83]
miR-375	*JAK2/STAT3*	Prevents the apoptosis of nasal mucosa cells [84]
miR-19a	*TGF-β1*	Attenuates allergen-induced suppression of *IL-10* in peripheral dendritic cells [83]
miR-26a	*SMAD2*, *SMAD3*	Involved in regulating TGF-β-dependent signaling pathways and reducing inflammatory reactions by either blocking *NF-κB* or promoting regulatory T cell responses [79]
miR-135a	*GATA-3*	Increases the levels of IL-4 and IgE in nasal mucosa and prevents mast cell activation/degranulation [85]
miR-221	*PI3K/AKT*	Promotes mast cell degranulation [86, 87]
miR-142-3p	*FceRI*	Enhances *FceRI*-mediated degranulation in mast cells [88]
miR-126-5p	*PI3K/AKT*	Promotes mast cell degranulation [89]
miR-146a-3p	*VAV3*, *PI3K/AKT/mTOR*	Promotes macrophage M2 polarization [90]
miR-18a	*CTGF* *TLR4*/NF-κB	Involved in the TGF pathway [79] Inhibits activation of the *TLR4*/NF-κB pathway [81]
MiR-29a-3p	*CTNNB1-VCL*	Promotes nasal epithelial barrier dysfunction [90]
MiR-223-3p	*FBXW7*	Regulates eosinophil degranulation and enhances inflammation [91]
MiR-193b-3p	*ETS1*	Suppresses inflammation by controlling TLR4 expression [92]
MicroRNA-29a	*FOS*	Downregulates *FOS* expression [93]
MicroRNA-150-5P	*EGR2*	Regulates Th1/Th2 cytokine expression by targeting *EGR2* in allergic rhinitis [94]
miR-224-5p	*GATA-3*	Assists in modulating the Th1/Th2 response [95]

Single-cell transcriptomics can reveal new epithelial cell types in AR. Daniel T. et al. [96] used single-cell RNA-seq and in vivo lineage tracing to study the cellular composition and hierarchical structure of the mouse tracheal epithelium. The study identified a novel gene (*FoxI1*, Forkhead Box I1) that plays a central role in the transcriptional program of lung ionophores. The investigators also identified columnar and mound cells with varying functions in AR, as well as disease-associated subpopulations of cluster and cup cells. That study revealed the complexity and diversity of the mouse tracheal epithelium, as well as the functional changes of different cell types under physiological and pathological conditions, which is important for understanding respiratory diseases and therapeutic development.

Recently, many studies have used transcriptomics to monitor the therapeutic effects of AR treatments [48, 97, 98]. Matthew et al. [99] recruited patients with AR and healthy volunteers to conduct a clinical trial with 16 weeks of treatment and showed that type 2 inflammation was the predominant type of disease pathophysiology in AR. After treatment with dupilumab, patients could significantly suppress the genetic characteristics manifested in the nasal allergen challenge when compared with those before treatment. Dupilumab inhibits the type 2 pathway and normalizes gene expression in nasal tissues.

Transcriptomic studies are important for the clinical study of childhood AR. Some phenotypes of AR are related to asthma, and timely diagnosis is helpful for disease stratification and targeted intervention. For example, changes in the expression of non-coding RNAs are related to inflammatory factors and cellular functions and can be used as potential diagnostic markers and therapeutic targets, but they are still in the exploratory stage. Although single-cell transcriptomics provides a powerful tool for research, the relevant results are still far from clinical translation. In addition, transcriptomics has also been used to monitor the effectiveness of AR treatment, such as the normalization of gene expression in nasal tissues by duplizumab treatment, which provides a basis for evaluating the effectiveness of treatment. At present, transcriptomic studies conducted for AR clinical research still have problems, such as scattered research results and a lack of systematic integration, which limits their comprehensive use for advancing precision diagnosis and treatment.

### Proteomics

Proteomics focuses on the quantitative analysis, post-translational modifications, and interactions of protein molecules. As the executors of biological functions, the expression of proteins directly reflects the metabolic state of cells. Proteomics originated with the rise of two-dimensional electrophoresis. As mass spectrometry technology continues to be updated and iterated, proteomics has transitioned from being a low throughput to a high-throughput tool, which allows for the systematic and comprehensive analysis of protein expression, structure, function, and interactions in specific cells, tissues, body fluids, and organisms [100, 101]. To identify biomarkers relevant to the early diagnosis and treatment of AR, many researchers have performed proteomic analyses of specimens from different sources, such as serum, nasal lavage fluid (NLF), and nasal mucosa from AR patients.

#### Research on Systemic Samples

Serum contains a large number of disease markers and is commonly used for proteomic analysis [102]. Chen et al. [103] collected serum from 45 patients with AR and 20 healthy control subjects and analyzed the samples by proteomics using iTRAQ technology. The results identified 133 differentially expressed proteins, including alpha-2-macroglobulin (A2M), fibrinogen, thrombospondin, coagulation factor XII, and coagulation factor III, which are mainly used in the analysis of serum because they are primarily involved in the coagulation pathway. This suggests that one important network circuit that controls the allergic inflammatory response in AR patients may be the blood coagulation pathway. One protein that is essential to the pathophysiology of AR is A2M, and its expression is regulated by STAT3 and positively correlated with IL17 expression and congestion symptoms. IL17 is expected to become an important target for the future treatment of AR. Choi et al. [104] used two-dimensional gel electrophoresis (2-DE) to show that the serum levels of lactoferrin (LTF) were lower in AR patients than in control subjects. That finding might be due to an increase in LTF in nasal secretions, which can ultimately lead to a decrease in serum LTF levels through local depletion or negative feedback regulation. While LTF protects AR against inflammation brought on by mast cells, the combined detection of serum LTF and dust mite sIgE levels can be used as an indicator for early monitoring of AR [105]. Blüggel et al. [106] analyzed the total proteins of CD4 + T cells in patients with AR before and after the pollen phase in a high-resolution 2D gel electrophoresis-based proteomic study and found that the levels of ankyrin 1, Nipsnap homologous protein 3A (NHP3A), and Glutamate-cysteine ligase regulatory proteins differed significantly, with NHP3A showing the most pronounced decrease. NHP3A plays a role in promoting vesicular transport and is involved in the oxidative stress response. That information provides a theoretical basis for investigating the molecular pathways and potential therapeutic targets of AR. Lee et al. [107] hypothesized that the RhoA/Rock signaling pathway is involved in tissue remodeling in AR and may be a target for intervention in AR. Approximately 30% of patients do not respond to AIT [108], and screening for biomarkers for early prediction of AIT efficacy can help patients receive the correct diagnosis and treatment. Ma et al. [109] used LC–MS/MS proteomics to compare various protein levels in patients who did and did not respond to AIT treatment and found that leukotriene A4 hydrolase levels were significantly elevated in the responder group, while those levels in the non-responder group showed no significant change, suggesting that serum could be used to predict the efficacy of AIT at an early stage. The levels of leukotriene A4 hydrolase in patients suitable for AIT were significantly increased after treatment when compared with those levels before treatment.

#### Research on Local Samples

Nasal secretions contain a variety of proteins, lipids, carbohydrates, and exfoliated cells, which can provide insights into the changes and roles of those components in diseases such as AR, and thus provide a scientific basis for diagnosis, treatment, and prevention of the disease. Kim et al. [110] performed liquid chromatography-mass spectrometry (MS)/MS in data-dependent acquisition (DDA) and data-independent acquisition (DIA) modes on nasal secretions from patients with AR and identified 2020 proteins. The proteins in nasal secretions were mainly antimicrobial proteins (e.g., lysozyme, lactoferrin), immunoglobulins (e.g., IgA, IgE, and IgG), and albumin, as well as kinin-releasing enzymes, antiproteases, and β-glucuronidase, which can function as enzymes, enzyme inhibitors or antioxidants, etc. [111]. Bryborn et al. [112] performed a 2-DE study which showed that S100A7 levels were significantly downregulated in the NLF of AR patients, making it the first biomarker to be identified in the nasal secretions of AR patients by use of proteomic techniques. S100A7 (Psoriasin protein) is an antimicrobial protein that is similar to other members of the S100 protein family. It specifically recognizes Ca-binding sites and targets chemotaxic CD4^+^ T lymphocytes, neutrophils, and granulocytes. Tomazic [113] performed a proteomic study of nasal mucus from patients with AR by using LC–MS/MS and found that apolipoprotein A-1 (APOA1), APOA2, A2M, α1-antitrypsin (SERPINA1), and complement protein C3 expression were all significantly increased in AR patients. Those proteins are mainly involved in the immune response. Ghafouri et al. [114] collected NLF from AR patients and analyzed the proteins by two-dimensional gel electrophoresis (2-DE) and matrix-assisted laser desorption/ionization time-of-flight mass spectrometry (MALDI-TOF MS) after trypsin cleavage. Two endogenous protease inhibitors, VEGP and cystatin, were found to be much less abundant in AR patients than in healthy controls. In contrast, the expression level of α−1-antitrypsin was higher in the AR patients when compared with controls, suggesting that endogenous antiproteases and innate immunity play an important role in AR. Corticosteroids and antihistamines are commonly used in the treatment of AR, and the efficacy of their use can be examined by investigating the proteomic properties of nasal NLF [115]. Wang et al. [48] performed a proteomics analysis which showed that the nasal secretions of AR patients before and after glucocorticoid treatment were significantly different with regard to their levels of oral mucus protein, APOA1, fibrinogen α-chain, histone D, and SERPINB3 proteins, which are involved in the glucocorticoid receptor pathway and the acute phase response pathway. Caillot et al. [116] found that salivary fetuin-A could serve as a biomarker for the early prediction of AIT efficacy by using the bidirectional difference gel electrophoresis technique. The nasal mucus, which is a direct reflection of pathophysiological processes occurring in the epithelium and is more readily available clinically, can be analyzed by proteomics to help study nasal secretions and their roles in healthy and diseased states. The contents of nasal secretions are potential biomarkers for use in new diagnostic and treatment methods for AR [117].

Proteins in serum can be used as indicators for early monitoring of AR and can predict the efficacy of AIT. However, most such studies have reported correlative findings, and the specific mechanisms and regulatory networks of those proteins are not yet fully understood. While many proteins in nasal secretions, such as antimicrobial and immunoglobulin proteins, show changes in patients with AR, the current studies are fragmented and lack systematic integration and large-scale validation. Although nasal mucosa proteomics research mostly relies on animal experiments, animal studies can show the differences in cell structure and the pathological changes which occur in the disease state. However, there are differences between animal models and the human body, and there are limitations to translating the results of animal studies to the clinic. Therefore, it is necessary to develop more accurate models.

### Metabolomics

Metabolomics focuses on the discovery of new biomarkers and the monitoring of therapeutic effects. Endogenous compounds (e.g., vitamins and amino acids) and exogenous chemicals (e.g., medications and poisons) are examples of metabolites, which are tiny molecules (< 1 kDa) that participate in chemical processes within organisms. Nuclear magnetic resonance and high-resolution mass spectrometry (MS) are the most common techniques used to characterize the metabolome [118, 119]. Specific metabolomic profiling of samples, including induced sputum, respiratory condensate, bronchoalveolar lavage fluid, serum, plasma, and lung tissues, reflect the different phenotypes, inflammatory patterns, and eventual outcomes of a disease. Metabolomic profiling can also help to discover pathophysiological mechanisms, subtype-specific biomarkers, and therapeutic targets [120].

#### Research on Systemic Samples

Recently, Chen et al. [121] performed a non-targeted metabolomics analysis of serum and feces from AR mice and healthy mice and discovered that when compared with a control group, the AR group’s levels of 16 blood metabolites, including dl-lactic acid, l-alanine, alpha-linolenic acid (ALA), and d-mannose, were considerably lower. Moreover, the levels of 11 other serum metabolites such as taurochromone deoxycholic acid, cholic acids, deoxycholic acid, uric acid, 3-phenylpropionic acid, and l-tryptophan were significantly increased in the AR mice. The researchers identified a total of 32 metabolites that were differentially expressed in the feces. In contrast to the control group, the AR group’s levels of 28 metabolites, such as purine, isobutyric acid, d-mannose, and propionic acid, were significantly higher, while the levels of four metabolites, such as ALA and deoxycholic acid, were significantly lower [121]. The production of key inflammatory mediators by the arachidonic acid (AA) metabolic network is considered to be a hallmark of various inflammation-related diseases [122]. Yuan et al. [122] used UPLC-MS/MS to study differences in serum metabolites between AR patients and healthy subjects. The analysis showed that 26 different metabolites (prostaglandin d2, 20-hydroxyleukotriene B4, linoleic acid, etc.)were significantly altered and 16 metabolic pathways (linoleic acid metabolism, AA metabolism, and tryptophan metabolism, etc.) were disturbed. In the AA/cyclooxygenase (COX) pathway, hematopoietic PGD synthase catalyzes the conversion of prostaglandin H2 (PGH2) to prostaglandin D2 (PGD2), which plays a role in modulating immune and inflammatory responses and activates eosinophils and basophils, making it a potential target and biomarker for use in treating allergic inflammation [123]. Sawane et al. [120] described an interesting phenomenon detected by a lipidomics analysis: the consumption of flaxseed oil, which is rich in ALA, helped to suppress AR. Flaxseed oil contains the unsaturated fatty acid eicosapentaenoic acid, which is an important member of the ω−3 family of fatty acids. The body converts eicosapentaenoic acid into 15-hydroxyeicosapentaenoic acid (15-HEPE). When a person with AR consumes flaxseed oil, the number of eosinophils in their body increases, and this increase promotes an accumulation of 15-HEPE in the nasal passages, which in turn helps to alleviate allergy symptoms. 15-HEPE is a newly discovered eicosapentaenoic acid-derived, eosinophil-dependent, anti-allergy substance, and causing an increase in its levels may be a novel approach for treating and preventing AR.

In addition, metabolomics can be used as a disease surveillance indicator to make judgments regarding the prognosis and severity of AR. Adamko et al. [124] recruited AR patients with proven allergy to ragweed and assessed the severity of their symptoms using the Total Nasal Symptom Score. By comparing differences in metabolite concentrations before and after a challenge, the researchers developed a metabolomic model based on partial least squares discriminant analysis that was effective for differentiating between patients with mild and severe AR. That proof-of-concept study suggested that metabolites in urine could be used for assessing the severity of AR. That finding might provide clinicians, allergists, and researchers with a new method for diagnosing AR, assessing treatment efficacy, and potentially providing clues for discovering novel therapeutic targets. Xie et al. [125] conducted a metabolomic analysis using ultra-high performance liquid chromatography-mass spectrometry (UHPLC-MS). The SLIT-treated patients were divided into an effective group and an ineffective group. An orthogonal partial least squares discriminant analysis (OPLS-DA) was performed to assess differences in metabolites between the two groups. The metabolites were mainly related to glycolysis and pyruvate metabolism, arginine and proline metabolism, and fatty acid metabolic pathways.

Metabolomics research in AR has mainly utilized mouse models and patient blood serum, feces, urine, and other systemic samples, but relatively few local tissues such as nasal mucosa and nasal lavage fluid have been used in metabolomics research. While the current research findings can help to reveal the metabolic mechanism of AR pathogenesis and provide clues for finding biomarkers, the data are susceptible to interference by many factors. Furthermore, individual variability is large, and there is a lack of in-depth mechanistic studies on how metabolites precisely regulate the process of AR. Further optimization of detection methods and validation studies of the accuracy of the models are needed to improve their reliability and stability before the models can be used for clinical applications. The identification of metabolites and their associated metabolic pathways makes it possible to understand the underlying pathogenic processes in patients with AR and enables further in-depth studies of AR based on metabolic evidence [126, 127].

### Microbiomics

The microbiome, often referred to as the second human genome, refers to the composition, function, and interactions of the host microbial community, which is a major component of the immune system. The microbiome is a functional entity that controls metabolism and regulates medication interactions [128]. Joshua Lederberg first used the term “microbiome” to “denote the ecological community of symbiotic, commensal, and pathogenic microorganisms that share our body space and are virtually ignored as determinants of health and disease.” The microbiome, which includes bacteria, fungi, and viruses, makes up 90% of the body’s total cell count, while what we commonly refer to as “body cells” (i.e., the cells that make up our organs and tissues) comprise only 10% of the total [129]. Although the widespread use of antibiotics and antimicrobials has raised concerns about their safety and potential side effects in some populations, the microbial community in the human body (including microorganisms in the gut and skin, etc.) has an extensive symbiotic relationship with the host and provides the host with genetic variety and gene functions that human cells have not acquired on their own.

#### Research on Systemic Samples

Increasing evidence suggests that patients with allergic diseases such as asthma [130, 131], food allergy [132], atopic dermatitis [133], and AR [134–138] have dysbiosis of the gut microbiota. Lee et al. sequenced 16S rRNA amplicons from bedroom dust samples obtained from 879 participants (mean age, 62 years) who vacuumed their homes to characterize bacterial communities. The study showed that several taxa from the phyla Cyanobacteria, *Anaplasma*, and *Clostridium* were more abundant in patients with asthma, allergic reactions, or hay fever, whereas allergic reactions were less frequent among participants who were exposed to families of the phylum Thicket [139]. The first indoor microbiome survey in an urban/rural context was a study that used birdshot macro genomic analysis. According to that study, urbanization and changes in indoor microbiome exposures were linked to greater prevalences of asthma and AR in urban regions when compared to rural ones [140]. Jin et al. [141] conducted a bidirectional Mendelian randomization analysis of the association between intestinal flora and atopic disease. The causal relationship was investigated and results showed that the class Coriobacteriia and its subgroups, the order Coriobacteriales and the family Coriobacteriaceae, all hindered AR development, while the family Victivallaceae was a risk factor for AR. In conclusion, AR is closely related to dysbiosis. In addition, some microorganisms play a therapeutic role in AR. Various studies have shown that the numbers of *Lactobacillus* were significantly reduced in an AR group and that supplements containing Lactobacilli or their metabolite lactic acid could alleviate the disease by improving the intestinal epithelial barrier, decreasing the levels of allergen-specific IgE, and increasing the levels of regulatory cytokine TGF-β [142–146] to alleviate allergic diseases. These findings indicate that Lactobacilli have a potential therapeutic value in AR. The results of microbiomic studies may provide innovative ideas for studying allergic diseases.

#### Research on Local Samples

Chua et al. [147] found that *A. tumefaciens* in the colon and lungs was associated with a higher prevalence of asthma than AR, because it promoted a significant expansion of Th2 cells and the infiltration of eosinophils and mast cells, which exacerbated airway inflammation in mice. That finding was further supported by Chen et al. [148] who showed that AR mice had a high relative abundance of pneumococci, which is considered to be one of the key genera that exacerbates allergic disease. According to a recent study, nasal epithelial epigenetic changes are the main cause of the correlation between nasal microbial abundance and an AR diagnosis. However, whether that association is also influenced by the presence of certain specific microorganisms (i.e., as a result of interactions between early exposure and immune system training, or other unknown factors) remains to be determined ^153^.

Although little is known about the function of such bacteria and more research is required, methods for modulating the numbers of specific bacterial taxa, such as increasing the number of beneficial bacteria and preventing the growth of harmful bacteria, may be used to prevent and treat allergic diseases such as AR.

## Multi-omics Finding Potential Targets

Combined multi-omic studies of AR from different perspectives can provide a more thorough and organized grasp of the pathophysiological mechanisms and individual differences of AR. Such studies can comprehensively analyze the genomic, transcriptomic, proteomic, and metabolomic multi-omics data of patients and facilitate the design of precise treatment regimens.

### Research on Systemic Samples

To gain insight into different candidate biomarkers for AR, Yuan et al. [122] analyzed the airway microbial communities and serum metabolomics of 28 AR patients and 15 healthy volunteers. Results showed that the structure of the microbiota in the AR cohort was altered by an abundance of one phylum (Actinobacteria phylum). Moreover, seven genera in the AR group were present at significantly higher numbers than in the healthy control group. Additionally, a total of 26 endogenous serum metabolites were identified as potential biomarkers, and the researchers calculated Spearman correlations between 15 microbial genera and 26 different metabolites. The AR-enriched genera were positively correlated with AR-enriched metabolites but negatively correlated with metabolites enriched in the healthy population, indicating that the increased abundance of those microbial genera in patients with AR was associated with increased concentrations of those metabolites. This suggests that those microbial genera play a role in the pathogenesis of AR or that their presence influences the production or metabolic pathways of the metabolites. A combined analysis of these microbiomics and serum metabolomics results produced highly correlated data that can be used for diagnosing AR.

To explore the key pathways associated with allergy formation, Sun et al. [149] performed an analysis based on network pharmacology and using a multi-omics approach. The analysis identified 39 important pathways in multiple histological datasets, of which 11 were ultimately critical pathways associated with allergy, including Th1 and Th2 cell differentiation, TLR cascade, and Th17 cell differentiation. These studies demonstrated that by integrating multi-omics data, key biomarkers and therapeutic targets for AR can be more comprehensively identified, laying a solid foundation for future therapeutic strategies.

### Research on Local Samples

Previous studies found that ginsenoside Rg3 is crucial for both preventing and treating allergic lower airway inflammation [150]. To investigate the role of Rg3 in allergic upper airway diseases such as AR, Liu et al. [151] collected specimens of nasal mucosa from control, AR model, and treatment mice and then screened them for differentially expressed genes and significantly altered metabolites by transcriptomic and metabolomic analyses. The results showed that nasal symptoms and inflammatory infiltration in mice were effectively ameliorated by intervention with Rg3. Rg3 can modulate an interaction network of 12 genes, 8 metabolites, and 4 pathways to exert anti-AR effects, inhibit inflammation development, and reduce oxidative stress. Leukotrienes and prostaglandins are implicated in the pathogenesis of AR. Tao et al. [152] identified and analyzed genes related to lipid metabolism pathways in AR and screened three diagnostic genes (*LPCAT1*, *SGPP1*, and *SMARCD3*) that showed significant variability between a control group and AR group. Those genes could possibly serve as biomarkers for AR.

Although AR shares many molecular mechanisms with other atopic diseases, omics studies have also revealed its unique features. First, AR primarily affects the local microenvironment of the nasal mucosa. Transcriptomic and proteomic studies have identified specific expression patterns of local inflammatory factors, chemokines, and immune cells in the nasal mucosa, while metabolomic studies have revealed distinct changes in metabolites within nasal secretions. Second, when compared with asthma or atopic dermatitis, the development of AR is more dependent on seasonal and specific allergens (e.g., pollen and dust mites). Epigenomic and metabolomic studies have further elucidated the dynamic changes in epigenetic modifications and metabolic responses triggered by allergen exposure. Moreover, although AR shares a Th2-mediated immune mechanism with other atopic diseases, the local synthesis of IgE and the activation of high-affinity IgE receptors highlight the immunological specificity of AR. Studies conducted on the nasal microbiome have also shown that the nasal microbial composition in patients with AR differs significantly from that of healthy individuals and patients with other atopic diseases (e.g., the airway microbiota in asthma or the skin microbiota in atopic dermatitis). Metabolomics has further revealed the regulatory roles of microbial metabolites in local inflammation. Finally, omics approaches have advanced the identification of therapeutic targets specific to AR, such as genomic and transcriptomic markers associated with the efficacy of antihistamines or immunotherapy. Moreover, metabolomics has provided insights into the impact of anti-inflammatory drugs on nasal metabolism. These findings have not only deepened our understanding of AR pathophysiology but also provided new directions for its precise diagnosis and treatment.

## Future Outlook

The European Academy of Allergy and Clinical Immunology Position Paper on the Application of Omics to Allergic Diseases provides comprehensive and authoritative guidance for applications in this field. It emphasizes the need for integrated multi-omics analyses and explores the potential of using omic markers for disease diagnosis. The paper also points out that the complexity and high dimensionality of omic data make data processing and analysis challenging, and that powerful bioinformatics tools and expertise will be needed in the future [153]. The application of multi-omic approaches in precision medicine research in AR is opening up new therapeutic and preventive perspectives. By integrating multi-omics data such as genomic, transcriptomic, proteomic, and metabolomic data, researchers can more comprehensively understand the complex biological mechanisms of AR and thus develop personalized treatment strategies [153].

**Fig. 2:**
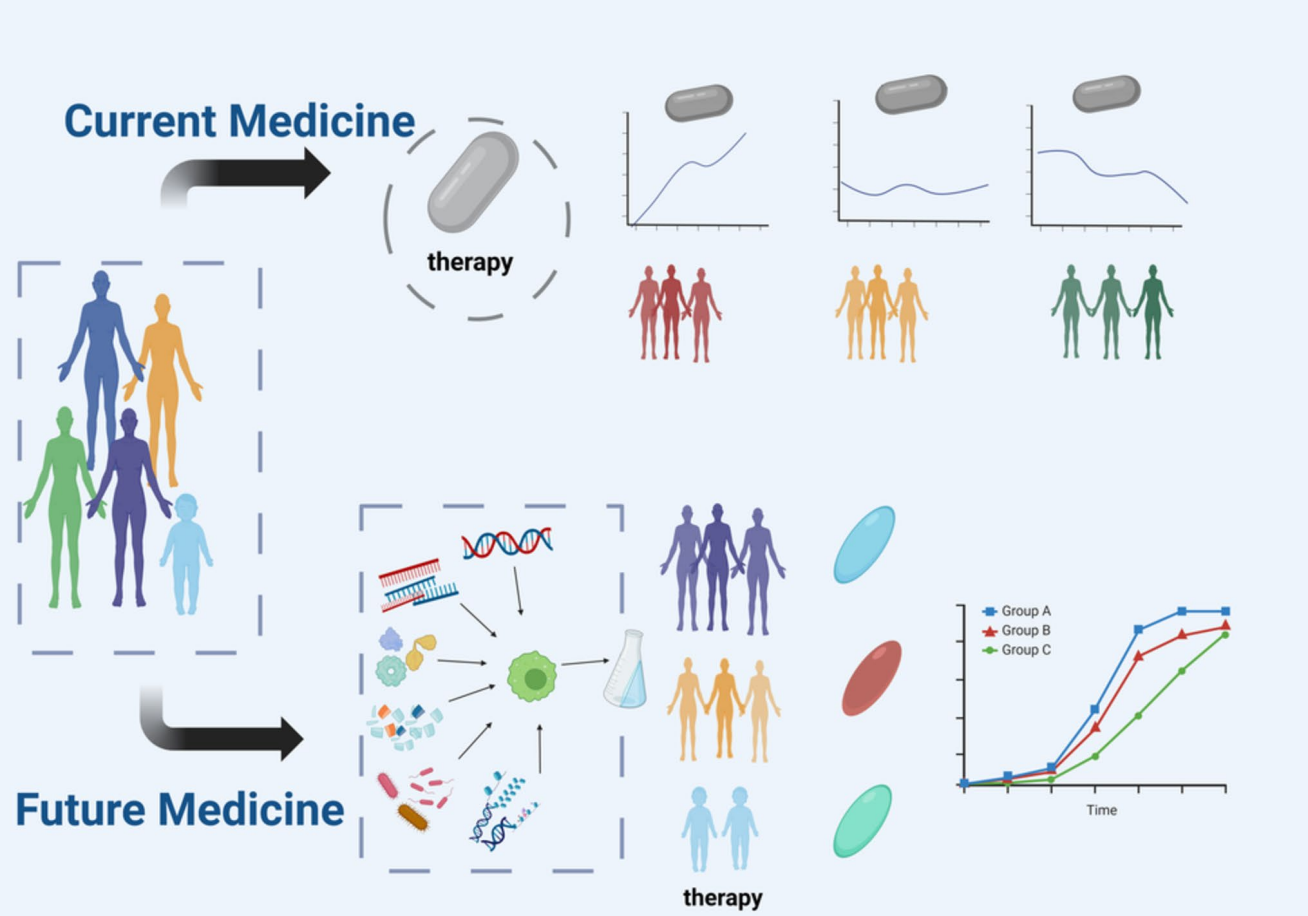
Precision diagnosis and treatment of allergic rhinitis. Traditional medicine relies on symptoms and experience and usually adopts standardized treatments based on a single approach. The treatment effects may fluctuate due to individual heterogeneity. Future precision medicine will integrate multi-omics data, deeply analyze the disease mechanisms of patients, focus on personalized customization, and tailor treatment plans based on individual biological information, making treatment more precise and effective

Future multi-omics studies will not only deepen our understanding of AR pathophysiology but also provide a strong basis for the development of more precise and effective diagnostic tools and therapeutic approaches (Fig. 2). First, continued technological advances will be of key importance for driving future research. With the continued development of advanced technologies such as next-generation sequencing, single-cell transcriptomics, spatial transcriptomics, spatial metabolomics, and mass spectrometry, it is anticipated that it will be possible to analyze and parse biological samples from patients with AR at a finer level. Second, biomarker discovery and validation will be an important direction for future research. Through multi-omics analysis, researchers can identify specific patterns of gene expression, protein expression, or metabolite changes that may serve as biomarkers of AR pathogenesis, progression, or therapeutic response. In addition, a comprehensive analysis based on multi-omics data will provide opportunities to develop new therapeutic targets. A deeper understanding of the molecular mechanisms of AR will allow for new pathological pathways or key molecules to be identified, which may become new targets for therapeutic interventions.

Multi-omics studies of AR face multiple challenges, and especially the increasing necessity for both standardized methods of sample collection and standardization of the samples. Given the heterogeneity of AR, samples from different patients may represent different stages or subtypes of the disease, further complicating a study. Thus, the importance of ensuring data comparability and reproducibility cannot be overstated, and studies must be conducted under tightly controlled conditions. In addition, differences in disease manifestations, clinical symptoms, and response to treatment among patients with AR highlight the complex molecular mechanisms underlying the disease. The heterogeneity of this disease requires not only the identification of shared molecular markers but also an in-depth understanding of the specific molecular features of the different subtypes or stages of the disease. To this end, larger studies and more detailed subgrouping are needed to reveal subtle differences in AR.

## Data Availability

No datasets were generated or analysed during the current study.
